# Modeling the Solubility of Phenolic Acids in Aqueous Media at 37 °C

**DOI:** 10.3390/molecules26216500

**Published:** 2021-10-28

**Authors:** Emilia Furia, Amerigo Beneduci, Luana Malacaria, Alessia Fazio, Chiara La Torre, Pierluigi Plastina

**Affiliations:** 1 Department of Chemistry and Chemical Technologies, University of Calabria, 87036 Arcavacata di Rende, CS, Italy; emilia.furia@unical.it (E.F.); luana.malacaria@unical.it (L.M.); 2 Department of Pharmacy, Health and Nutritional Sciences, University of Calabria, 87036 Arcavacata di Rende, CS, Italy; alessia.fazio@unical.it (A.F.); latorre.chiara@libero.it (C.L.T.); pierluigi.plastina@unical.it (P.P.)

**Keywords:** solubility, phenolic acids, activity coefficients, salting out constant, ionic medium

## Abstract

In this work, the solubility of vanillic, gallic, syringic, *p*-coumaric, ferulic and caffeic acids was determined at 37 °C under different conditions, namely pure water and two different ionic media, NaCl(aq) and NaClO_4_(aq), at different ionic strengths (i.e., 0.16, 0.50, 1.0, 2.0 and 3.0 M). The solubility in water of all the acids was found to be higher than that in both of the ionic media. Moreover, the solubility of hydroxycinnamic acids was lower than that of hydroxybenzoic acids. The activity coefficients of neutral species were calculated from these data; this knowledge is necessary when modeling the dependence of equilibrium constants on the ionic strength. Results obtained in this work can be useful for further studies regarding complex formation equilibria between these ligands and bioavailable metal cations.

## 1. Introduction

**Scheme 1:**
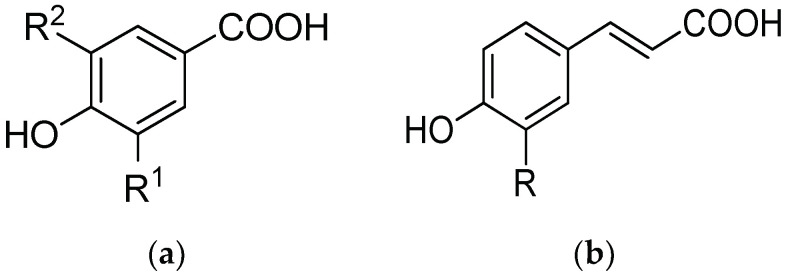
Chemical structure of (**a**) hydroxybenzoic acids (vanillic acid: R^1^ = OCH_3_, R^2^ = H; syringic acid: R^1^ = R^2^ = OCH_3_; gallic acid: R^1^ = R^2^ = OH); (**b**) hydroxycinnamic acids (caffeic acid: R = OH; ferulic acid: R = OCH_3_; *p*-coumaric acid: R = H).

A variety of phenolic compounds are currently among the most studied categories of natural antioxidants [1]. Phenolic acids contain one or more hydroxyl groups (polar part) attached to an aromatic ring (non-polar part) and are often found in plants as esters or glycosides [1,2,3,4,5,6]. Due to their ubiquitous presence in plant-based foods (fruit, vegetables, grain, tea, coffee, spices), the intake of phenolic acids is estimated as 25 × 10^−3^–1 g per day, depending on diet [7]. The interest in phenolic compounds lies mainly in their known health benefits, including their antioxidant activity and ability as free radical scavengers [8,9,10,11]. These properties give them great potential as active principles in the pharmaceutical industry as well as antioxidants in the food industry. For this reason, there is increasing interest in isolating these compounds from their natural matrices [12,13,14]. The efficient design of any extraction process requires the knowledge of the solute’s solubility. Aqueous solubility is a parameter of particular importance for assessing the environmental partitioning of different compounds. It has been reported that the low solubility of some solutes in water can be modified by the presence of co-solutes such as salts or by increasing the temperature [15,16,17,18,19,20,21]. Two phenomena related to solubility changes caused by the presence of co-solutes can be observed: salting-in and salting-out. In general, the solubility of a non-electrolyte can increase or decrease by the addition of an electrolyte, but the effect is dependent on the solvent salt used. Equation (1) relates the solubility of the neutral species, *S*°, to its activity coefficients, γ:

log γ = log (*S*°_0_/*S*°) (1)

where *S*°_0_ is the solubility at zero ion concentration. The activity coefficient of the neutral species is related to the molality, *m*, of the solvent electrolyte by Equation (2), also valid for weak electrolytes:

log γ = *k*·*m* (2)

where *k* is the salting-out (positive) or salting-in (negative) coefficient, also known as the Setschenow coefficient, which may depend on the ionic strength [22]. For a generic non-electrolyte, the effect depends upon the solvent salt; in general, for a given solvent salt, it depends upon the saturating non-electrolyte. According to Debye [23], the salting-out constant generally increases as the polar properties of the non-electrolyte decrease. This theory does not clarify how certain electrolytes salt-out a specified non-electrolyte while others do the opposite. A possible explanation has been proposed by Kruyt and Robinson [24]: in a solution of a non-electrolyte in water, the water dipoles are arranged around a molecule of the non-electrolyte, with their positive or negative end towards the non-electrolyte depending upon the polar properties of the latter. When an electrolyte is added to the solution, due to the hydration of the ions, less water is available to the saturating non-electrolyte and salting-out of the non-electrolyte is expected. There is an opposite effect: water molecules are organized in a manner differing to the arrangement about the non-electrolyte. The result of the approach of the ion to the non-electrolyte is the packing-in of water molecules about the non-electrolyte, i.e., salting-in. The present work represents the continuation of efforts concerning the evaluation of the effect of salt addition on the solubility of organic compounds [25,26,27,28,29]. We report here on the solubility at 37 °C of six phenolic acids of both subclasses, i.e., hydroxybenzoic and hydroxycinnamic acids, generically indicated as H*_n_*Ph—vanillic (VA), gallic (GA), syringic (SA), *p*-coumaric (*p*-CA), ferulic (FA) and caffeic (CafA) acids (Scheme 1)—in pure water as well as in aqueous solutions of different ionic strength in sodium chloride and in sodium perchlorate.

Previous literature data report on the solubility of these phenolic acids in pure organic solvents, in mixed organic–aqueous solutions [10,30,31,32,33,34,35,36,37,38,39,40,41,42,43], or in aqueous media but under experimental conditions far from biological. The interaction of these acids with aqueous media is important because such compounds display their antioxidant activity in the biological systems involving water as the natural solvent.

## 2. Results

**Figure 1:**
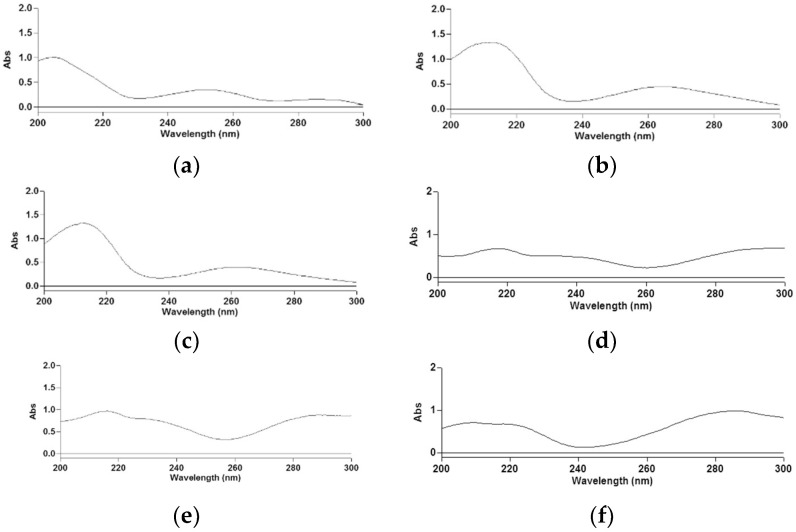
Absorption spectra of vanillic (**a**), syringic (**b**), gallic (**c**), caffeic (**d**), ferulic (**e**) and *p*-coumaric (**f**) acids.

The determination of phenolic acid solubility was achieved by measuring the absorbance values between 200 and 300 nm, every 1 nm, taking the ionic medium as a blank. Three replicates were run for each point. The typical spectra recorded for hydroxybenzoic (Figure 1a–c) and hydroxycinnamic (Figure 1d–f) acids are reported.

For the solubility determination of vanillic, syringic and gallic acids, peaks at 251, at 262 and at 258 nm, respectively, were considered. Concerning hydroxycinnamic acids, peaks at 287 for the quantitative determination of caffeic and ferulic acids and at 288 nm for *p*-coumaric acid were considered. The absorbance, *A*_λ_, of phenolic acids, generically H*_n_*Ph, was expressed according to Equation (3):

*A*_λ_ = *l* ε_λ_ [H*_n_*Ph] (3)

where *l* is the optical path and ε_λ_ is the molar absorptivity. The total solubility in pure water, *S*_T_°, as well as in aqueous solutions of different ionic strength in sodium chloride and in sodium perchlorate, generically *S*_T_, was deduced by interpolation on a calibration curve, based on standard solutions prepared in the range 2.0 × 10^−3^ and 25.0 × 10^−3^ molal for hydroxybenzoic acids and in the range 1.0 × 10^−3^ and 10.0 × 10^−3^ molal for hydroxycinnamic acids. In all cases, the correlation coefficient, *R*^2^, was ≥0.999 and the limit of detection was 0.50 × 10^−3^ molal. The total solubility values obtained at 37 °C are reported in Table 1, along with the outcomes of the statistical analysis.

**Figure 2:**
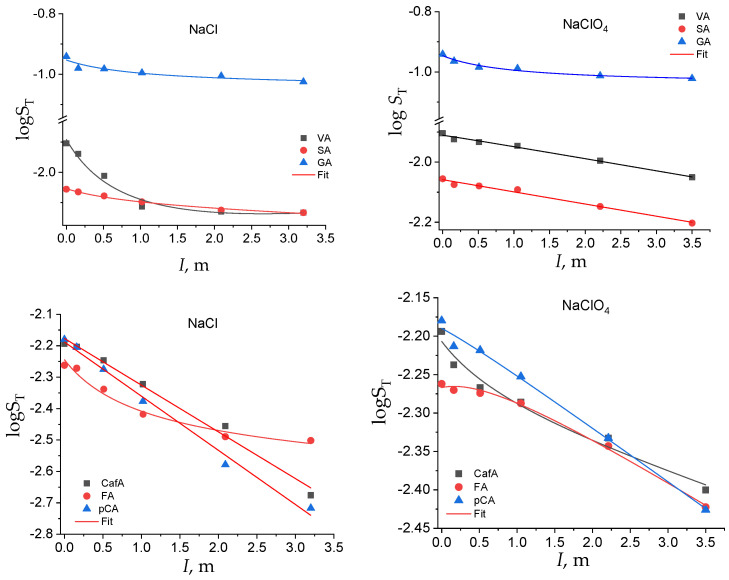
Total solubility in the logarithmic scale of the hydroxybenzoic and hydroxycinnamic acids as a function of the solvent salts and of the ionic strength.

As a general trend, the solubility in water of all the acids was higher than that in both ionic media at each of the electrolyte concentrations. The hydroxybenzoic acids were more soluble than the hydroxycinnamic ones and, among them, gallic acid showed the highest solubility, more than one order of magnitude higher. As expected, among the hydroxybenzoic acids, syringic acid was the least soluble, due to the presence of two methoxy groups, and the solubility of the hydroxycinnamic acids was generally lower than that of the hydroxybenzoic acids in all the media investigated. The salting-out effect is related to the strong tendency of ionic solutes to form hydration shells. In fact, as the concentration of the ionic salt increased, more and more water molecules were bound up in the hydration shells, and therefore the solubility decreased. The solubility trend of hydroxybenzoic and hydroxycinnamic acids as a function of the ionic strength can be better appreciated in Figure 2, in the two electrolyte media. 

In both sets of acids, the solubility was affected by the -OH and -OCH_3_ substituents on the phenyl moiety, with the hydroxy and methoxy groups contributing to an increase and a decrease in the solubility, respectively, especially at lower ionic strengths. The following order of solubility, that holds up to approximately *I* = 1 m, was found: GA > VA > SA and CafA > *p*-CA > FA. The effect of the substituents on the total solubility was more evident on the benzoic acid series. If we look at the dependence of *S*_T_ on the ionic strength for the benzoic acid series, the salting-out effect of the electrolyte was different for the two salts. In sodium chloride, the solubility dropped to low values in a smoothed way, at low ionic strength, and then it tended towards a plateau value. In perchlorate, instead, the solubility decreased almost linearly with *I* (except for GA). 

In the hydroxycinnamic acid series the solubility decreased quite linearly with the ionic strength, independently of the ionic medium used, except for the least soluble ferulic acid, which showed a nonlinear dependence of its *S*_T_ vs. *I* (Table S1 in Supplementary Material). The solid lines are the best fitting curves obtained by Equation (5) or by a line. The related fitting parameters are reported in Table S1.

The order of solubility traced above seems to be related to a polarity decrease in the acids as the hydroxyl groups are replaced by either a hydrogen or a methoxy group, and to the decrease in the capability of the molecule to make hydrogen bonds with water. This argument may explain why gallic acid has the highest solubility, almost one order of magnitude higher than the other acids of both series, at all ionic strengths, and why its solubility is slightly affected by the increase in the molality of the solvent salt.

However, this consideration is mostly important for the benzoic acids, while, in the hydroxycinnamic ones, the unsaturated alkyl chain plays a major role in determining the overall solubility behavior. 

In a recent computational study, it was shown that the free energy of solvation in water is negative for gallic and caffeic acids and positive for ferulic acid, with gallic acid having the most negative solvation free energy [44], indicating that the highest solubility of gallic acid is due to the high polar interaction density formed between the phenolic and carboxylic hydroxyl groups with water. In the same study, the authors showed also that the phenolic hydroxyl and the carboxyl functions tend to form hydrogen bonds with water, giving rise to a twist in the molecule structure close to the carboxyl group in the case of ferulic and caffeic acids. The effects of the phenyl hydroxy and methoxy substituents on the solid–liquid equilibrium of syringic and vanillic acids in water was recently investigated by the Abraham solvation model [45]. It was found that vanillic acid, with two hydroxyl substituents, had the highest value for the H-bond acidity (solute acidity descriptor), which described the tendency of the solute to form hydrogen bonds with its acid hydrogens, followed by syringic acid, which had one hydroxyl and two methoxy substituents. Moreover, the H-bond acidity of the carboxyl group increased by the electron-donating resonance effect of the methoxy substituents in the aromatic ring. In contrast, the H-bond acidity decreased due to the intramolecular hydrogen bond between two adjacent OH-OR substituents [46]. 

These acids may also form hydrogen bonds by sharing an oxygen lone electron pair of one of their substituents, i.e., they have hydrogen bond basicity. This is the highest for syringic acid due to the higher number of available lone electron pairs, even though intramolecular H-bonds may lead to a decrease in this parameter for vanillic acid [46]. Analogous results were obtained for ferulic and *p*-coumaric acids, the latter showing the highest value for the H-bond acidity parameter, which is partially reduced in ferulic acid due to the intramolecular hydrogen bond between the hydroxyl group and the methoxy group in the *meta* position. In contrast, the H-bond basicity character decreases from ferulic acid to *p*-coumaric acid, according to the increase in the number of hydrogen acceptors in the molecules [47]. 

Thus, the presence of hydroxyl groups increases the H-bond acidity, i.e., the strength of the hydrogen bonds formed by the donor hydroxyl groups with water, whereas the H-bond basicity descriptor, related to the strength of the hydrogen bonds formed by the acceptor groups in the molecules with water, increases with the number of methoxy substituents on the ring. On the other hand, when the hydroxy and/or the methoxy groups are absent, the above solute descriptors significantly decrease in value, such as in the case of veratric and cinnamic acids [46,47].

**Figure 3:**
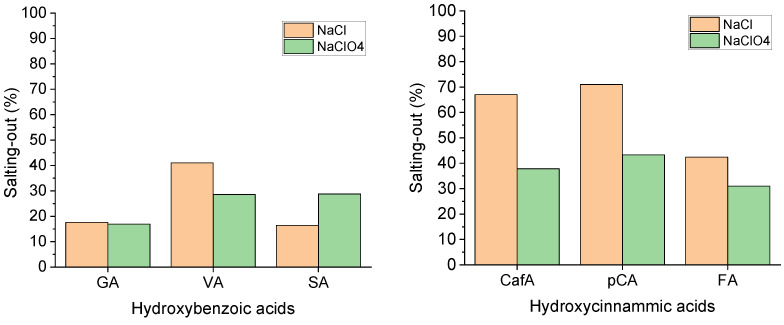
Salting-out effect calculated at the highest ionic strength for the two series of acids and in both electrolytes.

The largest salting-out effect calculated at the highest ionic strength is reported in Figure 3 in terms of the percentage decrease in the total solubility with respect to the value in pure water (Equation (4)).

(4) $$Salting-out\;\%=\left(S_{T}^{0}-S_{T}\right)\times 100/S_{T}^{0}$$

The salting-out effect is remarkable especially for the hydroxycinnamic acids, with values ranging from 40 up to 70% and mostly in NaCl. The strong decrease in the solubility with the ionic strength determines this result and highlights the low tendency of the hydroxycinnamic acids to interact with water, which becomes worse in the presence of highly polar salts such as sodium chloride. On the other hand, the salting-out effect seems to depend less on the type of electrolyte in the hydroxybenzoic acid series. It is interesting to note that in both ionic media and for both the acid series, the maximum salting-out occurs for the acids that have intermediate solubility in each series (i.e., vanillic and *p*-coumaric), which are the ones that respond mainly to ionic strength changes (Figure 2) mostly in NaCl medium.

## 3. Discussion

The knowledge of the activity coefficients of the neutral species is useful when modeling the dependence of equilibrium constants on the ionic strength [48,49]. For example, to evaluate the sequestering ability of these acids towards biological metal cations in a natural system, such as the ocean or biological fluids, the activity coefficients must be used to extrapolate their equilibrium constants at the infinite dilution reference state from those determined in constant ionic medium [50,51]. 

**Figure 4:**
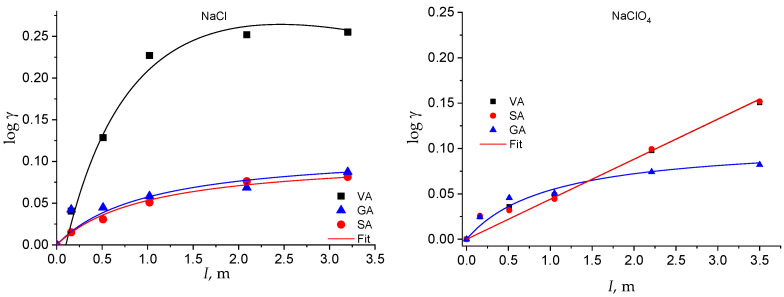
log γ of the hydroxybenzoic acids as a function of the solvent salt and of the ionic strength.

**Figure 5:**
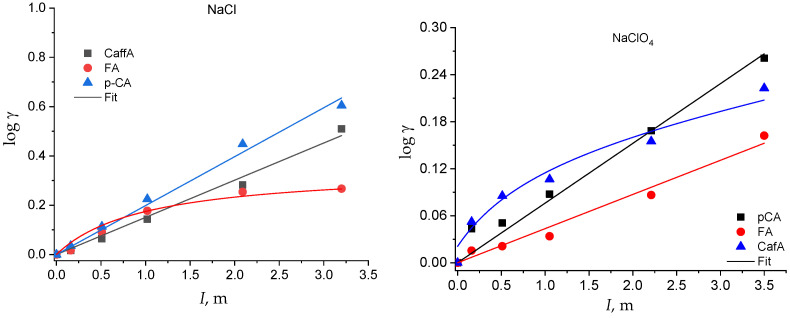
log γ of the hydroxycinnamic acids as a function of the solvent salt and of the ionic strength.

The activity coefficients of the neutral species as a function of *I* and of the solvent electrolyte are plotted in Figure 4 and Figure 5. The solubility of the neutral species at all the ionic strengths investigated is reported in Table S2 in the Supplementary Material.

The activity coefficient of the neutral species of all the acids increases with the ionic strength in both the solvent electrolytes (Figure 4 and Figure 5). However, some of them show a linear trend that can be well fitted by Equation (1), with Setschenow coefficients ranging from 0.7 up to 0.53 kg mol^−1^, while others show a nonlinear trend that is modeled by Equation (7) (Table S3 in Supplementary Material). In the latter cases, the Setschenow coefficient depends on the concentration of the solvent electrolyte and the log γ tends towards a plateau value for ionic strengths larger than around 2 molal. These data show that a salting-out effect in the total solubility corresponds to a salting-in effect on the activity coefficients, as already observed for other acids in aqueous solution [48].

Therefore, while, in NaCl medium, the solubility of the phenolic acids tends towards zero (and the activity coefficient tends towards infinity) at infinite *I*, in NaClO_4_, the solubility tends towards a minimum value (and the activity coefficient towards a maximum value) at very high *I*. This different behavior in the two electrolyte media is intriguing and deserves further study to be explained by changing the experimental conditions (i.e., the nature of inert salt) and extending the evaluation to other classes of analytes.

Table 2 collects literature data [6,30,32,34,39,41,43,46,52,53,54,55] for a comparison with our solubility results. All data are expressed as mass fraction (χ).

Our results are in line with previous literature data for vanillic, gallic, caffeic and *p*-coumaric acids. The agreement is also satisfactory for syringic and ferulic acids in pure water. There is less agreement with the data at 1 m NaCl and those reported previously in sulphate [30] and nitrate media [54], where the salting-out effect is higher than in chloride for syringic and vanillic acid and lower for gallic. This behavior has been already observed previously for syringic acid at room temperature [54] and clearly highlights the significant effect of the anion of the electrolyte salt. Moreover, the above discrepancies may be related to a substantial difference in the preparation of the saturated solutions: in our experimental approach, the direct contact of the analytes with the magnetic stirring is avoided and this is mainly reflected in the less soluble analytes.

## 4. Materials and Methods

### 4.1. Materials

All solutions were prepared by means of analytical-grade reagents and ultrapure water (Millipore MilliQ system). Vanillic (J&K Scientific, Beijing, China, 99%), syringic and gallic (Sigma Aldrich, St. Louis, MI, USA, ≥98% and ≥99%, respectively), *p*-coumaric (Sigma, St. Louis, MI, USA, ≥98%), ferulic and caffeic (Aldrich, St. Louis, MI, USA, 99%) acids were used without further purification. Sodium perchlorate and sodium chloride stock solutions were prepared and standardized according to previous works [56,57].

### 4.2. Solubility Apparatus and Procedure

The absorption spectra in the UV region were recorded with a Varian Cary 50 Scan UV–Visible spectrophotometer on a series of hydroxybenzoic and hydroxycinnamic acid test solutions, prepared by 100-fold dilution of 50 microliters of saturated solutions. These were prepared with a leaching apparatus suitable to prevent solid particles from coming into contact with the magnetic stirrer. To prevent grinding by the stirrer, hydroxybenzoic and hydroxycinnamic acids were wrapped in highly retentive filter paper (Whatman 42) bags. These were retained in glass cylinders containing pure water as well as sodium chloride and sodium perchlorate solutions at pre-established ionic strength values while continuously stirring with a magnetic bar. The cylinders were then placed in a thermostatic water bath at (37.0 ± 0.1) °C, and the hydroxybenzoic and hydroxycinnamic acid concentrations were monitored over time, until they reached a constant value, which usually occurred in around 4 days. Matched quartz cells of thickness 1 cm and 100 microliter volume were employed. The absorbance, *A*_λ_, was recorded to ±0.001 units. The formulations of the parameters and the acquisition of data were achieved with the aid of a computer connected to the instrument.

### 4.3. Thermodynamic Modeling

Total solubility data in the molal concentration scale were fitted by the smoothing empirical Equation (5):

(5) $$\log\left(S_{T}\right)=\log\left(S_{T}^{0}\right)+\left[a_{\infty }+\frac{a_{0}-a_{\infty }}{1+m}\right]m$$

where $S_{T}^{0}$ is the total solubility in pure water, and $a_{\infty }$ and $a_{0}$ are empirical parameters at infinite and at zero ionic strength, respectively. Equation (5) becomes linear for *a*∞ = *a*_0_ ≡ *a*.

The solubility of the neutral species, *S*^0^, was calculated by Equation (6), considering the deprotonation equilibrium of the phenolic acids (Equation (7)), behaving as a monoprotic acid with constant $K_{I}$ (Equation (8)):

(6) $$S^{0}=\frac{S_{T}\left[H^{+}\right]}{\left[H^{+}\right]+K_{I}}$$

(7) $$HA⇄H^{+}+A^{-}$$

(8) $$K_{I}=\frac{[H^{+}]\left[A^{-}\right]}{\left[HA\right]}\frac{\gamma_{H^{+}}\gamma_{A^{-}}}{\gamma_{HA}}$$

where, according to the specific ion interaction model (SIT) [58,59], the activity coefficients of the ionic species $\gamma_{\pm }$ and of the neutral one are given by Equations (9) and (10), respectively.

(9) $$log\gamma_{\pm }=-z_{\pm }^{2}\frac{0.4565I^{0.5}}{1+1.11I^{0.5}}+b_{\pm }I$$

(10) $$log\gamma_{HA}=kI$$

with $b_{+}=b\left(H^{+},Cl^{-}/ClO_{4}^{-}\right),\;b_{-}=b\left(A^{-},Na^{+}\right)\;$ and k is the Setschenow coefficient.

The dependence of the ionization constant of the acids on the ionic strength was explicitly considered in the calculation. Unfortunately, data on the protonation constant of these acids are only reported at a few ionic strengths and in the low molal range (up to 0.1 mol/kg) [60,61,62,63,64,65,66]. Therefore, here, the $K_{I}$ for each acid was measured at 0.16 mol/kg and 0.51 mol/kg and its values at higher ionic strength, up to 3.5 mol/kg, were estimated by the SIT model at 37 °C (Equations (8)–(11)).

(11) $$pK_{I}=pK_{0}-2D+(b_{+}+b_{-}-k)I$$

where $K_{0}$ is the value of $K_{I}$ at infinite dilution and $D=\frac{0.4565I^{0.5}}{1+1.11I^{0.5}}$ is the extended Debye term. $K_{0}$ was calculated by Equation (11) using the experimental values at *I* = 0.51 mol/kg.

In the SIT model (Equation (9)), the specific ion interaction coefficients relative to the proton with the anion of the electrolyte salt (i.e., Cl^−^, ClO_4_^−^) and that relative to the conjugate base of the acid (A^−^) with Na^+^ were kept constant in the iterations used for fitting the experimental data, respectively, at 0.12 (in NaCl)/0.14 (in NaClO_4_) [58] and 0.06 (in both media) [67]. For the specific ion interaction coefficient of A^−^ with Na^+^, we chose a value identical to that of the hydrogen salicylate ion with Na^+^, by considering the similarity of the anion structures. Figure S1 displays the dependence of the first ionization acidic constant vs. the ionic strength for all the investigated acids.

Equations (2) and (12), which consider the dependence of the Setschenow coefficients *k* on the ionic strength, were used to fit the activity coefficient data:

(12) $$\log\;\gamma =k_{\infty }+\left[k_{\infty }+\frac{k_{0}-k_{\infty }}{1+m}\right]m$$

where *k*_∞_ and *k*_0_ are the Setschenow coefficients at infinite and at zero ionic strength, respectively.

### 4.4. Statistical Analysis

All the experiments were performed in triplicate, and the data were expressed as means ± standard deviation. To test statistical differences among solubility values of each compound under different experimental conditions, data were evaluated with one-way ANOVA followed by Tukey’s multiple comparison test. A *p* value of < 0.05 was considered statistically significant.

## 5. Conclusions

The study of the solubility of benzoic and cinnamic acid derivatives is important for at least two aspects: one is on a fundamental thermodynamic basis, and concerns the knowledge of the solubility behavior at different temperatures and in different aqueous media, such as those containing electrolyte salts that may act as co-solvents. Calculation of the activity coefficients from the solubility values allows us to obtain a more complete picture of different aspects of the thermodynamic properties of hydroxybenzoic and hydroxycinnamic acids. Solubility measurements were analyzed to determine the Setschenow coefficients at infinite and at zero ionic strength and the solubility of the neutral species in pure water. Knowledge of these data simplifies the calculation of the activity coefficients of the charged and uncharged species. For instance, environmental partitioning of these acids in the aqueous phase is determined by their solubility in different conditions. Therefore, solubility studies of these compounds are of crucial interest for modeling the behavior of natural aquatic systems. The other aspect is of practical interest because the acids studied here are biologically active compounds that find applications in different industrial fields, such as pharmaceutical, cosmetic, food and biological wastewater treatment applications. In these cases, it is important to know the solubility in water and in other media in order to better design, for instance, processes aimed at their extraction from different matrices.

In this work, the solubility of hydroxybenzoic and hydroxycinnamic acids was studied in aqueous solutions of different ionic strengths at 37 °C. Here, we have verified that the higher polarity of the first class of phenolic acids reflects their higher solubility in aqueous solutions, independently of the ionic medium and the ionic strength. According to Kruyt and Robinson [24], this trend is due to the lower availability of the water molecules to the solvation of the phenolic acids because they are mainly involved in the ion’s hydration. The solubility of hydroxybenzoic acids decreases linearly when the inert salt is NaCl, while in NaClO_4_, the dependence on the ionic strength is smoothed. The behavior of hydroxycinnamic acids is different; in fact, the solubility of this class of acids decreases linearly with the ionic strength independently of the ionic medium. 

## Figures and Tables

**Table 1:** Total solubility at 37 °C of phenolic acids in water and in aqueous solutions of NaCl and NaClO_4_ at different ionic strengths.

$S_{T}\;10^{3},\;\mathrm{Molal}$						
	VA	SA	GA	CafA	FA	*p*-CA
**H_2_O**	^z^12.47 ± 0.01 ^a^	^y^8.8 ± 0.3 ^a^	^x^114.5 ± 0.3 ^a^	^w^6.4 ± 0.2 ^a^	^v^5.47 ± 0.01 ^a^	^w^6.61 ± 0.01 ^a^
***I* , molal NaCl**						
**0.16**	^z^11.52 ± 0.06 ^b^	^y^8.62 ± 0.03 ^ab^	^x^104.6 ± 0.3 ^b^	^w^6.26 ± 0.03 ^ab^	^v^5.35 ± 0.01 ^a^	^w^6.24 ± 0.01 ^a^
**0.51**	^z^9.74 ± 0.03 ^c^	^y^8.36 ± 0.06 ^ab^	^x^104.2 ± 0.4 ^bc^	^w^5.67 ± 0.04 ^bc^	^v^4.59 ± 0.09 ^b^	^w^5.31 ± 0.01 ^b^
**1.02**	^z^7.7 ± 0.6 ^d^	^z^7.99 ± 0.05 ^bc^	^y^101.1 ± 0.1 ^d^	^x^4.76 ± 0.01 ^d^	^w^3.82 ± 0.03 ^c^	^xw^4.19 ± 0.01 ^cg^
**2.09**	^z^7.42 ± 0.05 ^d^	^z^7.5 ± 0.1 ^cde^	^y^98.7 ± 0.2 ^e^	^x^3.50 ± 0.01 ^e^	^xw^3.24 ± 0.01 ^cd^	^w^2.64 ± 0.02 ^d^
**3.20**	^z^7.36 ± 0.03 ^d^	^z^7.36 ± 0.03 ^ce^	^y^94.4 ± 0.5 ^f^	^x^2.11 ± 0.03 ^f^	^w^3.15 ± 0.01 ^d^	^x^1.92 ± 0.08 ^e^
***I*, molal NaClO_4_**						
**0.16**	^z^11.92 ± 0.04 ^ab^	^y^8.42 ± 0.03 ^ab^	^x^104.6 ± 0.3 ^b^	^w^5.79 ± 0.04 ^bc^	^w^5.37 ± 0.01 ^a^	^w^6.12 ± 0.01 ^af^
**0.51**	^z^11.66 ± 0.04 ^b^	^y^8.33 ± 0.04 ^ab^	^x^103.7 ± 0.3 ^c^	^w^5.41 ± 0.01 ^c^	^w^5.32 ± 0.01 ^a^	^w^6.05 ± 0.05 ^af^
**1.05**	^z^11.33 ± 0.03 ^b^	^y^8.10 ± 0.09 ^bd^	^x^102.6 ± 0.8 ^g^	^w^5.18 ± 0.06 ^cd^	^w^5.16 ± 0.01 ^ab^	^w^5.59 ± 0.04 ^bf^
**2.21**	^z^10.1 ± 0.1 ^c^	^y^7.12 ± 0.02 ^e^	^x^97.1 ± 0.1 ^h^	^w^4.65 ± 0.04 ^d^	^w^4.54 ± 0.07 ^b^	^w^4.65 ± 0.04 ^c^
**3.50**	^z^8.9 ± 0.3 ^e^	^y^6.27 ± 0.01 ^f^	^x^95.1 ± 0.1 ^i^	^w^3.98 ± 0.02 ^e^	^w^3.78 ± 0.03 ^cd^	^w^3.75 ± 0.07 ^g^

The values with different superscripts (a, b, c, d, e, f, g, h and i) in the same column are significantly different (*p* < 0.05). The values with different superscripts (v, w, x, y and z) in the same row are significantly different (*p* < 0.05).

**Table 2:** Solubility of phenolic acids, as mass fraction (χ), in water and in 1 molal NaCl at 37 °C from this work and from the literature.

χ‧10^3^														
Phenolic Acids	This Work	Ref. [6]	Ref. [30]	Ref. [32]	Ref. [34]	Ref. [39]	Ref. [41]	Ref. [43]	Ref. [46]	Ref. [52]	Ref. [53]	Ref. [54]		Ref. [55]
**H_2_O**														
**VA**	0.23								0.22 ^b^			0.24 ^c^ 0.26 ^d^		0.24
**GA**	2.10		1.33	2.79	2.16	2.50	2.66					1.31 ^c^ 1.38 ^d^		1.40
**SA**	0.16	0.36	0.84						0.22 ^b^			0.79 ^c^ 0.92 ^d^		0.85
**CafA**	0.12			0.19										
**FA**	0.10			0.14					0.11 ^b^	0.113 ^c^; 0.079 ^d^	0.0807 ^c^; 0.109 ^d^			0.90
***p*-CA**	0.12	0.086							0.12 ^b^					
**χ‧10^3^**														
**Phenolic Acids**	**This Work**													
	**1 m NaCl**											**1 m KNO_3_**	**1 m NaNO_3_**	
**VA**	0.14											0.19 ^c^ 0.23 ^d^	0.17 ^c^ 0.20 ^d^	
**GA**	1.85		0.98 ^a^									1.27 ^c^ 1.34 ^d^	1.15 ^c^ 1.21 ^d^	
**SA**	0.14											0.76 ^c^ 0.88 ^d^	0.71 ^c^ 0.88 ^d^	
**FA**	0.07													

^a^ Calculated at 37 °C in 1 m Na_2_SO_4_; ^b^ Calculated from the experimental solubility values at 40 °C expressed as g solute/100 solvent by the equation: $\chi =\frac{{\left(\frac{m}{M}\right)}_{acid}}{{\left(\frac{m}{M}\right)}_{acid}+{\left(\frac{m}{M}\right)}_{water}};$ ^b^ Measured at 35 °C; ^c^ Measured at 35 °C; ^d^ Measured at 40 °C.

## Data Availability

Not applicable.

## Supplementary Materials

The following are available online, Figure S1. Dependence of the ionization constant on the ionic strength in the two salts media. Experimental values (symbols) and values modeled by the SIT theory (lines) with the equation 11. For all of the fitting, an adjusted *R*^2^ > 0.998 and a reduced χ^2^ as low as 10^−5^ were obtained. Table S1. Smoothed total solubility data as a function of the ionic strength in NaCl and NaClO_4_. Table S2. Solubility of the neutral species at 37 °C of phenolic acids in water and in aqueous solutions of NaCl and NaClO_4_ at different ionic strength. The uncertainties represent standard deviation. Table S3. Setschenow coefficients of hydroxybenzoic and hydroxycinnamic acids in NaCl and NaClO_4_.

## Sample Availability

Samples of the compounds are not available from the authors.
