# Supplementary material for: Modeling the Solubility of Phenolic Acids in Aqueous Media at 37 °C
Source: Molecules. 2021 Oct 28;26(21):6500. doi: 10.3390/molecules26216500 (PMC8587167; doi:10.3390/molecules26216500)
Supplement: Supplementary file 1 [file molecules-26-06500-s001.zip › molecules-1432716-supplementary.pdf]

Modelling the solubility of phenolic acids in aqueous media at 37 °C

Emilia Furia[1], Amerigo Beneduci[1,*], Luana Malacaria[1], Alessia Fazio[2], Chiara La Torre[2] and Pierluigi Plastina[2]

[1] Department of Chemistry and Chemical Technologies, University of Calabria, Arcavacata di Rende (CS), Italy; emilia.furia@unical.it (E.F.); luana.malacaria@unical.it (L.M.)

[2] Department of Pharmacy, Health and Nutritional Sciences, University of Calabria, Arcavacata di Rende (CS), Italy; alessia.fazio@unical.it (A.F.); chiara.latorre@unical.it (C.L.T.); pierluigi.plastina@unical.it (P.P.)

* Correspondence: amerigo.beneduci@unical.it (A.B.)

Table S1. Smoothed total solubility data as a function of the ionic strength in NaCl and $NaClO_4$[a].

	$\log(S_T^0)$	a_0	a_∞ a (for linear fit)	Reduced $\chi^2\times10^4$	Adj. R^2
**NaCl**					
VA	-1.89 (0.02)	-0.43 (0.062)	0.03 (0.02)	4	0.96135
SA	-2.054 (0.004)	-0.078 (0.01)	-0.009 (0.004)	0.18	0.98234
GA	-0.95 (0.01)	-0.09 (0.04)	-0.002 (0.001)	3	0.97818
CafA	-2.18 (0.01)		-0.145 (0.008)	0.1	0.9903
FA	-2.315 (0.009)		-0.050 (0.005)	5	0.98635
p-CA	-2.19 (0.01)		-0.172 (0.008)	4	0.99048
	$\log(S_T^0)$	a_0	a_∞ a (for linear fit)	Reduced $\chi^2\times10^4$	Adj. R^2
**$NaClO_4$**					
VA	-1.911 (0.006)	-0.04 (0.02)	-0.041 (0.006)	0.4	0.98455
SA	-2.054 (0.004)	-0.078 (0.01)	-0.009 (0.004)	0.18	0.98234
GA	-0.946 (0.004)	-0.096 (0.008)	0[b]	0.3	0.9598
CafA	-2.21 (0.01)	-0.13 (0.04)	-0.03 (0.01)	0.2	0.96067
FA	-2.267 (0.004)	-0.53 (0.03)	-0.071 (0.008)	1	0.98718
p-CA	-2.187 (0.005)		-0.067 (0.003)	0.9	0.98977

[a]Values in parentheses are the standard deviations of the data as determined by the fit; [b]The parameter a_∞ has been kept fixed to zero during the iteration because it is more than one order of magnitude smaller than a_0.

Table S2. Solubility of the neutral species at 37°C of phenolic acids in water and in aqueous solutions of NaCl and $NaClO_4$ at different ionic strength. The uncertainties represent standard deviation.

	S^0 10^3. mol kg^{-1}					
	**VA**	**SA**	**GA**	**CafA**	**FA**	***p*-CA**
***I* mol kg^{-1} NaCl**						
**0**	12±3	8±2	113±15	6.1±0.9	5±1	6.2±0.9
**0.16**	11±2	8±2	102±13	5.8±0.8	5±1	5.8±0.8
**0.51**	9±1	8±2	102±13	5.3±0.7	4±1	4.8±0.6
**1.02**	7±1	7±2	98±12	4.4±0.6	3±1	3.7±0.5
**2.09**	7±1	7±2	96±12	3.2±0.4	3±1	2.2±0.3
**3.20**	7±1	7±2	92±12	1.9±0.3	3±1	1.5±0.2
***I* mol kg^{-1} $NaClO_4$**						
**0**	12±3	8±2	113±16	6.1±0.9	5±1	6.2±0.9
**0.16**	11±2	8±1	106±15	5.4±0.8	5.0±0.7	5.6±0.8
**0.51**	11±2	8±1	101±14	5.0±0.7	4.9±0.7	5.6±0.8
**1.05**	11±2	8±1	100±14	4.8±0.7	4.8±0.7	5.1±0.7
**2.21**	10±1	7±1	95±13	4.3±0.6	4.2±0.6	4.2±0.6
**3.50**	9±1	6±1	93±13	3.7±0.6	3.6±0.5	3.4±0.5

Table S3. Setschenow coefficients of hydroxybenzoic and hydroxycinnamic acids in NaCl and $NaClO_4$[a].

	$\boldsymbol{k_0}$<br>***k* (for linear fit)**	$\boldsymbol{k_\infty}$	**Reduced $\chi^2 \times 10^4$**	**Adj. R^2**
**NaCl**				
VA	0.57 (0.01)	-0.05 (0.06)	82	0.97706
SA	0.106 (0.003)	0[b]	0.11	0.98948
GA	0.11 (0.01)	0[b]	2	0.92014
CafA	0.151 (0.005)		5	0.99278
FA	0.35 (0.02)	0[b]	4	0.97025
p-CA	0.199 (0.006)		4	0.99486
	$\boldsymbol{k_0}$<br>***k* (for linear fit)**	$\boldsymbol{k_\infty}$	**Reduced $\chi^2 \times 10^4$**	**Adj. R^2**
**$NaClO_4$**				
VA	0.044 (0.002)		1	0.96640
SA	0.044 (0.002)		5	0.98505
GA	0.108 (0.005)	0[b]	0.4	0.95551
CafA	0.17 (0.05)	0.02 (0.01)	3	0.95770
FA	0.044 (0.002)		0.82	0.97809
p-CA	0.076 (0.004)		2	0.97401

[a]Values in parentheses are the standard deviations of the data as determined by the fit; [b]The parameter $\boldsymbol{k_\infty}$ has been kept fixed to zero during the iteration because it is more than one order of magnitude smaller than $\boldsymbol{k_0}$.

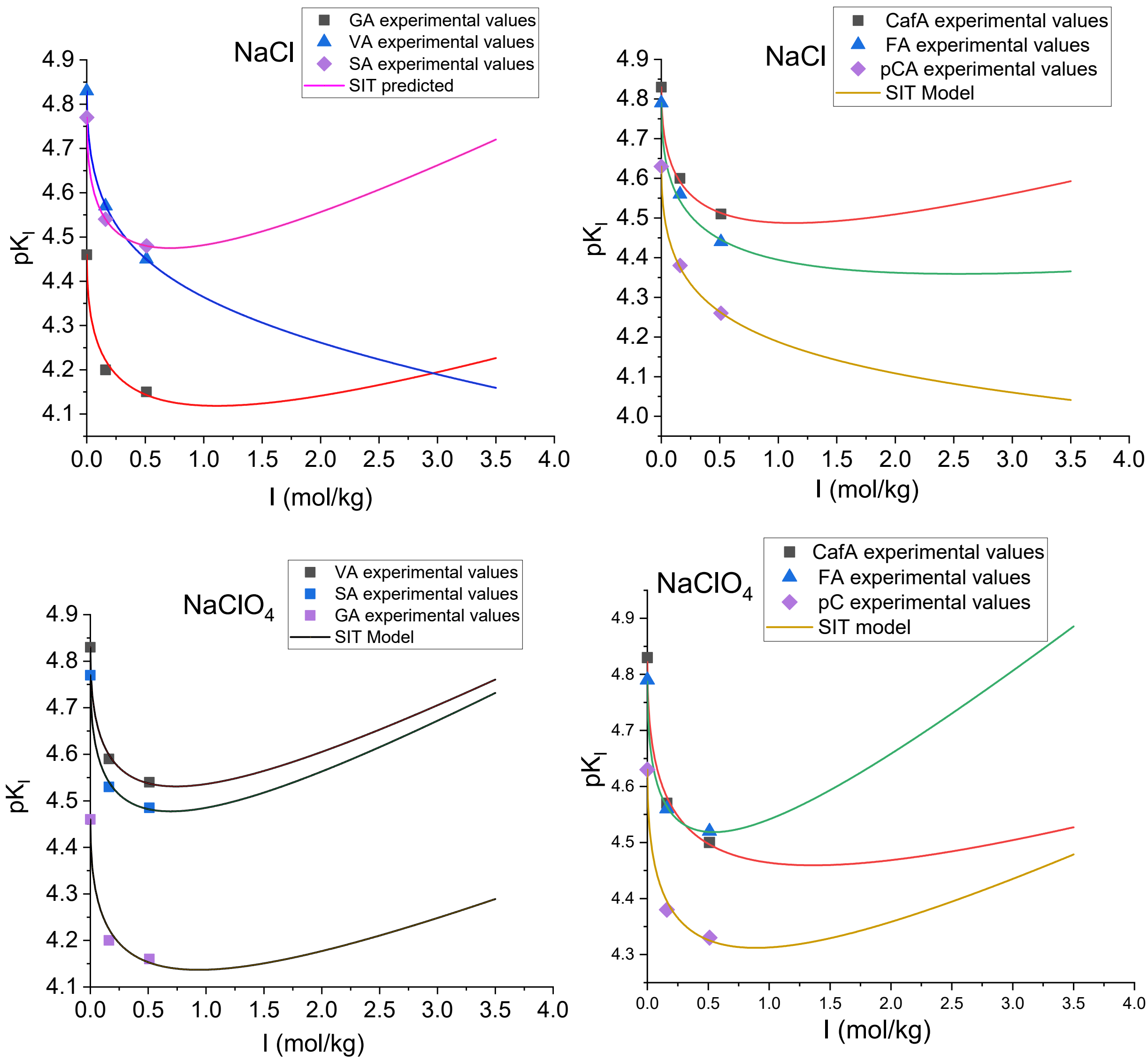

Figure S1. Dependence of the ionization constant on the ionic strength in the two salts media. Experimental values (symbols) and values modeled by the SIT theory (lines) with the equation 11. For all of the fitting, an adjusted $R^2 > 0.998$ and a reduced χ^2 as low as 10^{-5} were obtained.
